# Adipose Tissue Redox Microenvironment as a Potential Link between Persistent Organic Pollutants and the 16-Year Incidence of Non-hormone-Dependent Cancer

**DOI:** 10.1021/acs.est.0c08180

**Published:** 2021-06-28

**Authors:** Vicente Mustieles, Francisco M. Pérez-Carrascosa, Josefa León, Theis Lange, Jens-Peter Bonde, Celia Gómez-Peña, Francisco Artacho-Cordón, Rocío Barrios-Rodríguez, Rocío Olmedo-Requena, José Expósito, José J. Jiménez-Moleón, Juan P. Arrebola

**Affiliations:** † Center for Biomedical Research (CIBM), University of Granada, Instituto de Investigación Biosanitaria Ibs GRANADA, 18016 Granada, Spain; ‡ Consortium for Biomedical Research in Epidemiology and Public Health (CIBER Epidemiología y Salud Pública, CIBERESP), 28029 Madrid, Spain; § Department of Radiology and Physical Medicine, University of Granada, 18016 Granada, Spain; ∥ Instituto de Investigación Biosanitaria Ibs GRANADA, 18012 Granada, Spain; ⊥ Radiotherapy and Oncology Department, University Hospital Virgen de las Nieves Granada, 18014 Granada, Spain; # Unidad de Gestión Clínica de Aparato Digestivo, Hospital Universitario San Cecilio de Granada, 18016 Granada, Spain; ¶ Section of Biostatistics, Department of Public Health, University of Copenhagen, DK-1165 Copenhagen, Denmark; ∇ Department of Occupational and Environmental Medicine, Bispebjerg University Hospital, Bispebjerg Bakke 23F, 2400 Copenhagen NV, Denmark; ○ Universidad de Granada, Departamento de Medicina Preventiva y Salud Pública, 18016 Granada, Spain

**Keywords:** oxidative stress, persistent organic pollutants, organochlorine pesticides, polychlorinated biphenyls, cancer

## Abstract

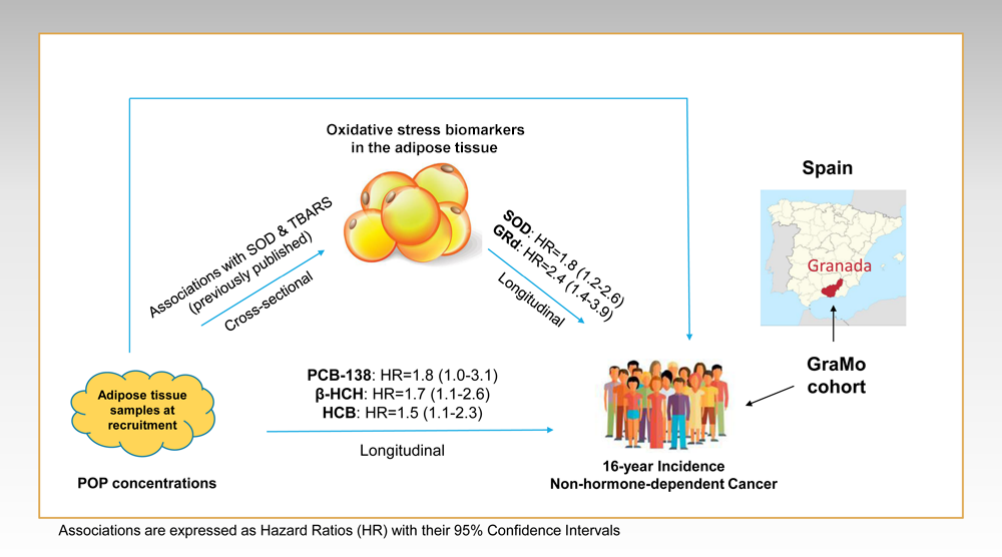

We aimed to assess the relationships among the adipose tissue’s (AT) oxidative microenvironment, *in situ* accumulated persistent organic pollutant (POP) concentrations, and cancer development. POP and oxidative stress levels were quantified in AT samples from 382 adults recruited within the GraMo cohort (2003–2004) in Granada (Spain). The 16-year cancer incidence was ascertained by reviewing health/administrative databases. Cox-regression models and mediation analyses were performed. The enzymes superoxide dismutase (SOD) and glutathione reductase (GRd) were positively associated with the risk of non-hormone-dependent (NHD) cancer [adjusted hazard ratio (HR) 1.76; 95% confidence interval (CI): 1.17, 2.64 and HR 2.35; 95% CI: 1.41, 3.94, respectively]. After adjustment for covariates, polychlorinated biphenyl-138 (PCB-138) (HR 1.78; 95% CI: 1.03, 3.09), β-hexachlorocyclohexane (β-HCH) (HR 1.70; 95% CI: 1.09, 2.64), and hexachlorobenzene (HR 1.54; 95% CI: 1.02, 2.33) were also positively associated with the risk of NHD cancer. Although confidence intervals included the null value, probably because of the modest number of cancer cases, we observed a potential mediation effect of SOD and GRd on the associations between β-HCH and the risk of NHD tumors (percent mediated = 33 and 47%, respectively). Our results highlight the relevance of human AT’s oxidative microenvironment as a predictor of future cancer risk as well as its potential mediating role on POP-related carcinogenesis. Given their novelty, these findings should be interpreted with caution and confirmed in future studies.

## Abstract 2

Human adipose tissue’s oxidative microenvironment may predict future cancer risk and play a role on POP-mediated carcinogenesis.

## 1 Introduction

Cancer constitutes a major public health problem and one of the leading causes of death worldwide.^1,2^ Environmental and lifestyle factors account for an important proportion of the cases, and up to 50% of all cancers could be prevented.^3^ In addition to dietary habits, physical activity, smoking, and alcohol consumption, environmental pollution also contributes to this global burden.^4^

Previous epidemiological studies have reported associations between obesity and the risk of several cancer types.^5^ The dysfunctional adipose tissue (AT) is associated with a chronic state of increased oxidative stress and low-grade inflammation,^6,7^ which can lead to insulin resistance, as well as the secretion of adipokines and inflammatory cytokines, in turn contributing to tumor development.^7−9^ Chronic redox imbalance leads to the production of free radicals and reactive metabolites, the so-called reactive oxygen species (ROS), and sustained exposure to ROS may cause significant damage to diverse biological structures.^10^ Oxidative stress is linked to cancer initiation and progression by increasing DNA mutations or inducing DNA damage, genome instability, and cell proliferation.^11,12^ Additionally, oxidative stress can modulate the expression of more than 500 different genes, including inflammatory cytokines, chemokines, growth factors, and cell cycle regulatory molecules.^11,12^

A sometimes overlooked but important fact is that environmental pollutants, especially the most lipophilic and persistent ones, tend to accumulate in human AT, constituting an internal exposure source for chemical mixtures.^13^ At the same time, AT is a target itself of these pollutants, which may exert a local effect by interfering with the lipid metabolism, insulin sensitivity, and the redox microenvironment.^14−16^ Therefore, AT has been proposed as an ideal matrix for understanding how accumulated environmental exposures can influence its local environment, eventually leading to a potential increased risk of cancer.^13,17^

Persistent organic pollutants (POPs), including organochlorine pesticides (OCPs) and polychlorinated biphenyls (PCBs), are highly lipophilic chemicals that persist in the environment, accumulate in organisms, and biomagnify in the food chain.^18^ Humans are thus daily exposed through diet, especially fatty animal food consumption.^19−21^ Although the use of most OCPs and PCBs in agricultural, industrial, and commercial applications has been banned or severely restricted in most countries, these chemicals are still present in the environment and, therefore, detected in virtually all human populations and wildlife.^22^

Experimental studies have confirmed the ability of OCPs and PCBs to interact with biological functions through different action mechanisms.^13^ These POP families can bind to a number of nuclear receptors, including estrogenic, peroxisome proliferator-activated receptor γ (PPAR-γ), and aryl hydrocarbon receptors.^14,18^ POPs can also interfere with epigenetic regulation processes, inducing altered DNA methylation and micro-RNA expression.^23−26^ Moreover, increasing evidence highlights their potential to induce oxidative stress and inflammation in both experimental^18,27−29^ and epidemiologic studies.^15,30,31^

POP exposure in the general population, although at relatively low levels, is increasingly linked to chronic diseases including metabolic syndrome and cancer.^13,32,33^ Epidemiologic studies have suggested a role of POP exposure in the etiology of some of the most prevalent cancer locations including liver,^34^ non-Hodgkin lymphoma,^35^ colorectal,^36−38^ prostate,^39,40^ and breast cancer^41−43^ among other types.^33^ In contrast, other studies and meta-analyses did not find enough evidence to support these associations^44−47^ or have been more cautious in the light of inherent methodological limitations.^48^

While most previous studies characterized POP exposure in blood, AT is probably the most adequate matrix for assessing long-term exposure to POP mixtures and an interesting tissue to investigate subclinical effects.^13,49^ Our preliminary findings in the GraMo cohort at year 9 of the follow-up suggested potential associations between concentrations of certain POPs in AT and total cancer risk.^33^ Accumulated POP concentrations in this same cohort were later found to influence the AT oxidative microenvironment.^15^ Animal models have suggested a relevant role of AT oxidative stress in the etiology and progression of cancer.^6,11,50^ A number of POPs can exert both endocrine and non-endocrine modes of action^18^ and have been associated with both hormone-dependent (HD) and non-hormone-dependent (NHD) tumors.^47,48^ Although increased oxidative stress may influence most cancer types, we hypothesize that this mechanism may be more relevant for NHD tumors compared to HD malignancies since the latter are primarily influenced by known endocrine mechanisms.^51,52^ Therefore, this study aimed to shed light on the POP exposure-AT redox microenvironment-cancer triad by investigating the following: (i) whether the AT oxidative microenvironment is associated with total, NHD, and HD cancer incidence; (ii) the relationship between long-term accumulation of POPs and the 16-year cancer risk; and (iii) whether oxidative stress may mediate POP-cancer associations.

## 2 Materials and Methods

### 2.1 Study Population

The GraMo cohort is a hospital-based study aimed to characterize human exposure to environmental factors and their contribution to the development of chronic diseases in adults from Granada Province, Southern Spain. The study design, recruitment, and methods have been extensively described elsewhere.^53,54^ In brief, study participants were recruited between July 2003 and June 2004 in two public hospitals: San Cecilio University Hospital in the city of Granada (240,000 inhabitants) and Santa Ana Hospital in the town of Motril (50,000 inhabitants). Patients undergoing scheduled non-cancer-related surgery were asked to donate an AT sample during the surgery, together with a morning 12 h-fasting blood sample the same day of the intervention, following the standard surgery protocols of the hospitals. All the study participants were users of the public health system. Inclusion criteria were as follows: age over 16 years, absence of previous cancer, non-prescription of hormonal therapy, and residence in one of the study areas for at least 10 years. Reasons for surgery comprised a total of 70 different health issues, including hernias, gallbladder diseases, varicose veins, and other conditions. All participants signed their informed consent, and the study was approved by the Ethics Committee of Granada (Comité de Ética de la Investigación Biomédica de la Provincia de Granada, 08/2016).

**Figure 1:**
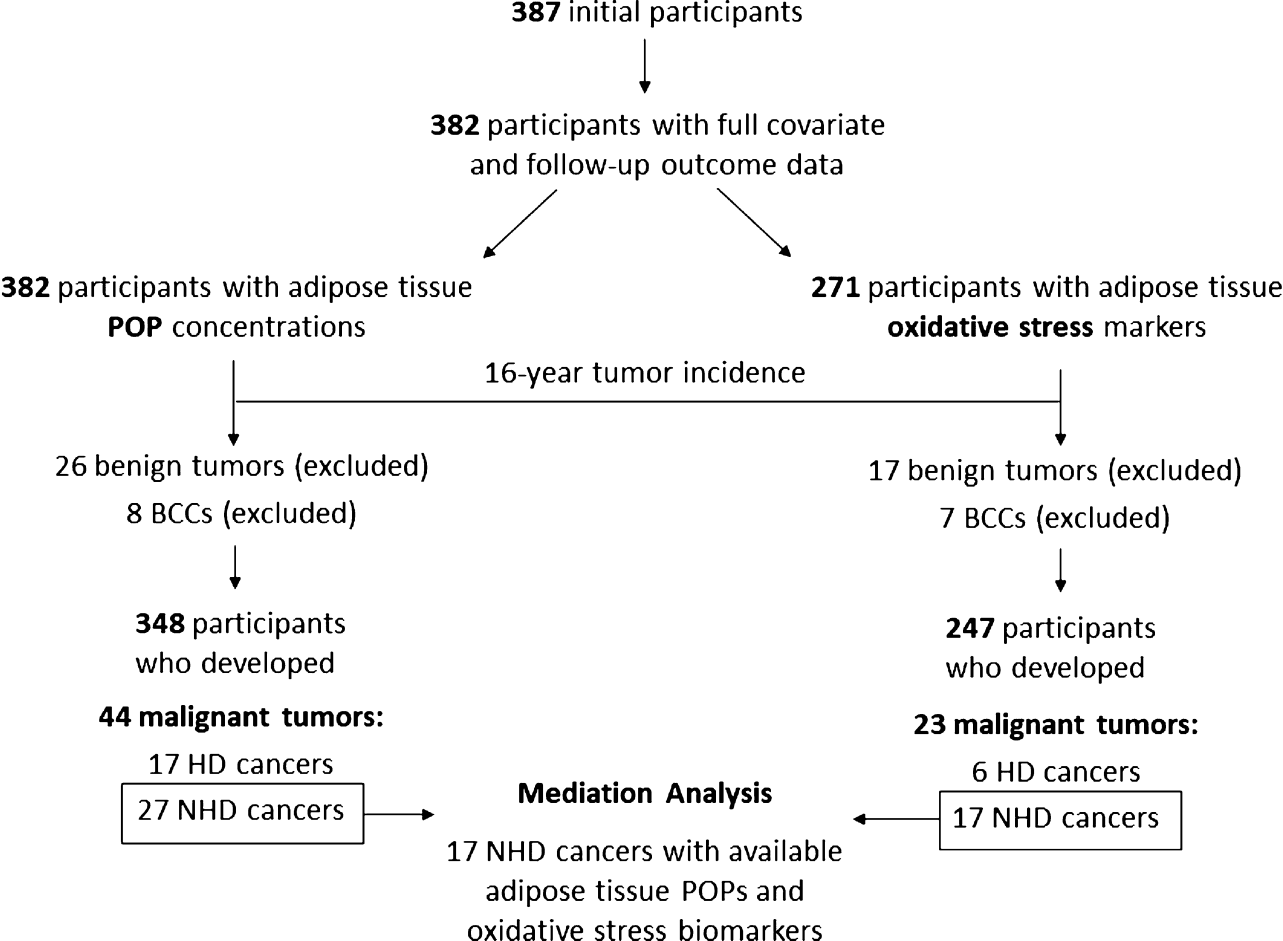
Flowchart describing incident cases in the study population. Note: out of 348 participants with POPs in AT and clinical data, a subset of 247 participants also had AT oxidative stress biomarkers based on sample availability.

Out of 409 individuals who were invited, 387 agreed to participate in the study. Of these, we excluded participants with a previous cancer diagnosis, benign tumors, and basal cell carcinomas (BCCs) or limited/incongruent clinical information in the reviewed health databases at follow-up, leaving a final sample size of 348 participants for which POP concentrations in AT, clinical information, and covariates were available. Data on biomarkers of AT oxidative stress, clinical information, and covariates were available for 247 participants. The main reason for this smaller subset with oxidative biomarkers was AT sample availability. Figure 1 shows a flowchart of the study population analyzed. When participants with both POPs and oxidative stress biomarkers (*n* = 247) were compared with those that only had POP measurements (*n* = 101), no significant differences were observed for sociodemographic or POP concentrations, with the exception of a slightly higher proportion of males and a higher body mass index (Supporting Information Table S1). Main characteristics of the study population are presented in Table 1.

**Table 1:** Cancer Events, Localization, and Classification According to Hormone versus Non-Hormone Dependence^a^

			ICD-10	*n*	*n* %
non-hormone-dependent	BCCs	other malignant and unspecified malignancies	C44	8	15.4
	non-BCCs	colon	C18	4	7.7
		lung	C34	4	7.7
		rectum	C20	3	5.8
		larinx	C32	2	3.8
		skin	C43	2	3.8
		leukemia	C90	2	3.8
		buccal mucosa	C14	1	1.9
		malignant stomach neoplasms	C16	1	1.9
		liver	C22	1	1.9
		GIST abdominal	C26.9	1	1.9
		connective tissue	C49	1	1.9
		bladder	C67	1	1.9
		brain	C71	1	1.9
		thyroid	C73	1	1.9
		follicular lymphoma	C82	1	1.9
		multiple myeloma	C90	1	1.9
hormone-dependent		breast	C50	7	13.5
		prostate	C61	5	9.6
		uterus body	C54	4	7.7
		testicle	C62	1	1.9
total number of malignancies including BCCs				52	100%
total number of malignancies excluding BCCs				44	84.6%

a BCCs: basal cell carcinomas; GIST: gastrointestinal stromal tumor.

### 2.2 Exposure Assessment

Samples of AT (5–10 g) were intraoperatively collected by hospital surgeons and immediately coded and stored at −80 °C until chemical analysis. Chemical extraction with *n*-hexane was performed on 200 mg of AT, and the resulting solution was then purified through 2 g of alumina in a glass column. All extracts were stored in glass tubes at −80 °C.

POPs were quantified by high-resolution gas chromatography coupled with a mass spectrometry detector in the tandem mode using a Saturn 2000 ion trap system (Varian, Walnut Creek, CA). A 2 m × 0.25 mm silica capillary column was used for the analysis (Bellefonte, PA) coupled with a 30 m × 0.25 mm analytical column (Factor FOUR VF-5MS, Varian Inc., Walnut Creek, CA). The limit of detection (LOD) was 0.01 μg/L for all POPs under study. POP concentrations <LOD were assigned a random value between 0 and the LOD using the = RAND excel function of Microsoft Excel (v.2102). Residues of the following chemicals were quantified: *p*,*p*′-dichlorodiphenyldichloroethylene (*p*,*p*′-DDE, the main metabolite of the pesticide DDT), hexachlorobenzene (HCB), dicofol, α- and β-hexachlorocyclohexane (α- and β-HCH, respectively), and PCB congeners −138, −153, and −180. C13-labeled *p*,*p*′-DDE, caffeine, and 3′-fluoro-2,4,4′-trichlorobiphenyl (PCB 28F) were used as internal standards. Inter- and intraday variability was <20%. For the quality control, laboratory-fortified matrix samples at different concentrations were used. POP recoveries in AT ranged between 90 and 98%, and blank samples were tested to avoid potential external contamination (always yielding a negative result).^55^ Lipid content in AT samples was quantified gravimetrically, including a homogenization step of 100 mg of AT with 5 mL of chloroform/methanol/hydrochloric acid (20:10:0.1) and posterior acidification with hydrochloric acid 0.1 N before collecting and weighing the organic phase. Lipid-basis POP concentrations were calculated by dividing crude AT concentrations by total AT lipid content, expressed as nanograms of POPs per gram of lipid (ng/g lipid). Further details on the validation of the analytical method can be consulted elsewhere.^53−57^

### 2.3 Oxidative Stress Biomarker Measurements

Oxidative stress biomarkers were analyzed using commercially available kits (Enzo Life Sciences, Inc., Farmingdale, NY, USA) in an automatic microplate reader (TRIAD MRX II series, Dynex Technologies Inc., Chantilly, Virginia, USA). AT samples were slowly thawed on ice and repeatedly washed with cold PBS to remove blood clots and other debris. Tissues were then homogenized in the appropriate buffer at the proportion specified by each kit using a pestle.^58^ The following biomarkers were assessed: superoxide dismutase (SOD) activity, heme oxygenase-1 (HO-1), glutathione peroxidase (GPx) and glutathione reductase (GRd) activities, total glutathione, reduced (GSH) and oxidized (GSSG) glutathione, thiobarbituric acid reactive substances (TBARS), and 8-hydroxydeoxyguanosine (8OHdG). Additional methodological details can be consulted elsewhere.^15,58^

### 2.4 Outcome Assessment and Classification

In order to minimize information loss, the 16-year cancer incidence was ascertained by reviewing both administrative and clinical databases. First, we performed an exhaustive and individualized review of clinical records, at both primary and specialized care. We further consulted complementary databases, including diagnostic tests, drug dispensing, and laboratory tests. Finally, the abovementioned data were contrasted with those from external administrative databases including death and residence registers. Overall, 15 different databases were consulted. The follow-up time began on the recruitment date and continued until the diagnosis of cancer or the patient’s death. If the patient did not experience any of these events, this follow-up period ended on 31 August 2019, although the cohort remains under study.

In the current study, cancer was defined as the diagnosis of any malignant neoplasm, and tumors were classified according to the International Statistical Classification of Diseases and Related Health Problems, 10th Revision (ICD-10, codes C00–C97, except non-melanoma skin cancer).^59^ Benign tumors (*n* = 19) were excluded from the analyses due to their uncertain evolution (specific locations are presented in Table S1 of the Supporting Information). BCCs were also excluded from the main analyses (*n* = 8) since BCCs constitute a group with particular etiologic and defined risk factors.^60^ BCC is the most common cancer in humans, mostly arising on the sun-damaged skin in the head and neck, and is usually a slow-growing tumor rarely associated with metastases or fatal outcomes.^60^ Therefore, in the current work, we considered the relationship between AT’s oxidative microenvironment and three outcomes: (i) total cancer risk based on our previous pilot study,^33^ (ii) the risk of NHD cancer, and (iii) the risk of HD cancer. The associations explored between POPs and cancer were guided by the results between oxidative stress and cancer risk, given that we aimed to explore POP-cancer associations that could be mediated by *in situ* oxidative stress.

### 2.5 Covariates

Data on sociodemographic characteristics, lifestyle, and health status were obtained in face-to-face interviews conducted by trained personnel at the time of recruitment during the hospital stay. The questionnaire was designed and validated in a previous investigation.^61,62^ Participants’ weight and height were measured, and body mass index (BMI) was calculated as weight/height squared (kg/m^2^). A subject was considered a smoker or alcohol consumer with any level of daily tobacco (≥1 cig/day) or weekly alcohol (≥1 drink/week) consumption. Residence in the city of Granada at the time of the surgery was considered “urban”, and residence in the coastal area of Motril was considered “semi-rural”. This covariate also accounts for the hospital of recruitment (Granada vs Motril). Education level was classified as incomplete primary studies, primary studies (schooling from 6 to 12 years of age), and secondary or higher education (schooling from 12 to 16 years of age or superior).

### 2.6 Statistical Analysis

Descriptive analysis included the calculation of medians and 25/75th percentiles for the interval variables and percentages for the categorical variables.

The magnitude of associations between AT concentrations of POPs or AT levels of oxidative biomarkers and the 16-year cancer incidence was evaluated using Cox-regression models with time-to-events as the time variable, calculating hazard ratios (HRs) with their corresponding 95% CIs. Estimations of time-to-events were based on the dates of recruitment, diagnosis, and end of follow-up. Data on participants who died before the observation of the study outcome were censored; therefore, only their disease-free time was considered in the analyses. Natural-log (ln) transformed lipid-basis POP concentrations (ng/g lipid) were used as the independent variables (for POPs whose detection rate ranged between 86 and 100%). Dicofol (% >LOD = 19.4%) and α-HCH (% >LOD = 22.2%) were entered as dichotomous variables (≥LOD/<LOD). The shape of the relationships between individual POP concentrations and the outcome was evaluated by using Generalized Additive Models (GAM).

Mediation analyses were conducted using the methodology described by Lange and colleagues.^63^ HRs and their 95% CIs were calculated for the direct, indirect, and total effects. The percentage mediated was calculated as = indirect effect/(direct effect + indirect effect) × 100. Mediation analysis estimates the proportion of a statistical relationship between a given exposure and outcome that occurs through a change in the mediator. The natural direct effect estimates the change in cancer risk estimates for each log-unit increase in POP concentrations, when the mediator (i.e., oxidative stress) remains unaltered. Thus, it represents the proportion of the POP-cancer association attributable to POPs acting through mechanisms different from oxidative stress. The natural indirect effect (i.e., mediated effect) estimates the change in risk estimates when POP concentrations are held unaltered, and a given oxidative stress marker increases by the amount it would have changed had the POP concentrations increased by 1 log unit. In other words, it evaluates the contribution of POPs on cancer that goes through a certain oxidative stress marker. The total effect represents the relationship between the exposure and the outcome without accounting for any mediator. For the mediation analyses, only the oxidative markers and POP congeners that were associated with the outcome were considered.

Covariates were selected based on those variables whose inclusion in any model produced changes >10% in β-coefficients and/or those reported as relevant confounders in previous studies. Thus, all models were adjusted for the same set of covariates: age (years), sex (male/female), BMI (kg/m^2^), place of residence (urban vs semi-rural), and education (lower than primary education, primary education, or higher than primary). We additionally adjusted for smoking (yes/no) and alcohol consumption (yes/no) since both substances are recognized risk factors for a diversity of cancer types and interfere with cytochrome P450 enzymes, which also participate in the metabolism of POPs.^64,65^

Data were stored and processed using R statistical computing environment v3.0,^66^ and the following packages were used: medflex,^67^ mice,^68^ survival,^69,70^ and Amelia.^71^ The significance level was set at *p* ≤ 0.05, and all tests were two-tailed. We interpreted our results considering their internal validity and coherence, magnitude of effect estimates, and previous biological and epidemiological evidence rather than solely depending on p-values and statistical significance.^72^

### 2.7 Sensitivity and Stratified Analyses

The potential modifying effect of age, sex, BMI, education, place of residence, smoking habit, and alcohol consumption on the associations found was studied by entering their product terms (POP levels * each potential modifier) in the models. Based on previous results,^33^ we performed sex-stratified models to test if the POP-cancer associations differed between males and females. A conservative approach was followed in the main models, in which BMI was considered as a confounder. However, BMI could also be regarded as an intermediate variable between POPs and cancer. Thus, an additional sensitivity analysis was performed excluding BMI from the models to test its impact on POP-cancer associations and on the mediation analyses. We also tested the robustness of the main results between oxidative stress and POP biomarkers with cancer risk, further adjusting the models for surgery reasons [hernias (41%), gallbladder diseases (21%), varicose veins (12%), and other conditions (26%)].

## 3 Results

### 3.1 Characteristics of Study Participants

Out of the 387 initial participants with AT at recruitment, 26 (6.8%) developed a benign tumor and were excluded from the analysis (Figure 1 and Supporting Information, Table S2). A total of 52 (13.6%) incident cancer cases were registered over the 16-year follow-up (Table 1). Of these, 8 BCCs were excluded, leaving a final number of 44 malignancies: 27 NHD and 17 HD cancers (Figure 1). Out of the 348 participants studied, 43 (12.4%) non-cancer-related deaths occurred during the follow-up, and their data were censored. The median follow-up time, including censored and non-censored data, was 185.7 months.

The sociodemographic characteristics of the study population and AT POP concentrations are reported in Table 2. In comparison with participants who did not develop cancer during the follow/up (*n* = 304), cancer cases (*n* = 44) were significantly older (median age: 60 vs 48 years) and had a significantly higher BMI (median BMI: 27.5 vs 26.3 kg/m^2^). Among participants with a cancer diagnosis during the follow-up, there was a higher proportion of alcohol consumers (59.1 vs 51.0%), individuals with less than primary education (34.1 vs 26.3%), and urban residents (56.8 vs 50.7%). No substantial differences were observed according to sex or smoking status. When sociodemographic characteristics were stratified by sex, we observed a higher number of urban residents, smokers, and alcohol consumers in men versus women (Supporting Information Table S3).

**Table 2:** Sociodemographic Characteristics and AT POP Exposure Levels in the Study Population (*n* = 348)^a^

	total study participants (*n* = 348)^b^		participants free of cancer (*n* = 304)		participants with any type of malignancy (*n* = 44)		
	*n*	%	*n*	%	*n*	%	*P*-value^c^
Sex = male	175	50.3	173	50.0	23	52.3	0.872
education							0.301
primary uncompleted	95	27.3	80	26.3	15	34.1	
primary	155	44.5	140	46.1	15	34.1	
secondary or higher	98	28.2	84	27.6	14	31.8	
residence							0.519
urban	179	51.4	154	50.7	25	56.8	
semi-rural	169	48.6	150	49.3	19	43.2	
alcohol consumer (=yes)	181	52.0	155	51.0	26	59.1	0.337
smoker (=yes)	113	32.5	100	32.9	13	29.5	0.732

	DF (%)	median (P25, P75)	median (P25, P75)	median (P25, P75)	
age (years)		50.5 (36.0, 63.0)	48.0 (34.0, 61.0)	60.0 (52.5, 72.0)	<0.001
BMI (kg/m^2^)		26.5 (23.8, 29.4)	26.3 (23.6, 29.4)	27.5 (24.5, 30.1)	0.052
PCB-138 (ng/g)	86	82.4 (30.6, 138.4)	70.2 (24.9, 126.7)	110.9 (86.6, 208.0)	<0.001
PCB-153 (ng/g)	92	218.2 (136.4, 361.9)	204.3 (119.1, 332.2)	310.4 (233.8, 513.1)	<0.001
PCB-180 (ng/g)	90	178.5 (102.8, 296.2)	171.2 (97.0, 278.3)	260.9 (183.7, 372.7)	<0.001
*p*,*p*′-DDE (ng/g)	100	94.7 (32.9, 210.4)	78.1 (30.7, 190.0)	175.7 (107.4, 312.6)	<0.001
HCB (ng/g)	91	14.6 (5.0, 39.6)	*12.6 (4.8, 35.9)*	32.7 (13.8, 72.1)	<0.001
β-HCH (ng/g)	84	10.5 (3.7, 21.2)	9.4 (2.8, 19.3)	19.2 (11.3, 28.0)	<0.001

	*n*	%	*n*	%	*n*	%	
dicofol (>LOD)	71	20.4	63	20.7	8	18.2	0.842
α-HCH (>LOD)	70	20.1	55	18.1	15	34.1	0.025

a Data are presented as frequencies and percentages for categorical variables or as median (percentile 25, percentile 75) for continuous variables. BMI (body mass index); PCB 138, 153, and 180 (polychlorinated biphenyls 138, 153, and 180); *p*,*p*′-DDE (*p*,*p*′-dichlorodiphenyldichloroethylene); β-HCH (β-hexachlorocyclohexane); HCB (hexachlorobenzene); α-HCH (α-hexachlorocyclohexane); DF (detection frequency).

b A total of 348 participants with available persistent organic pollutant (POP) concentrations in AT after excluding benign tumors and basal cell carcinomas (BCCs). During the 16-year follow-up period, 304 participants remained free of cancer, and 44 developed any type of malignancy.

c *P*-value for the comparison between cancer and non-cancer cases. Fisher’s exact test and Mann–Whitney’s *U* for categorical and continuous variables, respectively.

Regarding the distribution of baseline POP concentrations in AT, with the exception of dicofol, all concentrations were significantly higher in incident cancer cases compared to the remaining participants (Table 2). Women presented higher concentrations of *p*,*p*′-DDE, HCB, α-HCH, and β-HCH than men (Supporting Information Table S3). AT POP concentrations in the GraMo cohort and their comparison with other contemporary populations have been previously discussed.^53,54,57^

Certain oxidative stress biomarkers in AT were higher in cancer cases than in the rest of participants, especially SOD (median levels: 13.7 vs 8.67 U/mL) and GRd (median levels: 0.15 vs 0.10 U/mL) (Table 3), while total glutathione levels were lower (7.46 vs 17.3 nmol/mL). With the exception of GSSG and the GSSG/GSH ratio, AT oxidative biomarkers did not substantially differ according to sex (Supporting Information Table S4). The levels and predictors of AT oxidative stress biomarkers in the GraMo cohort have been previously described.^15,58^

**Table 3:** AT Levels of Oxidative Stress Biomarkers in the Study Population (*n* = 247)^a^

	total study participants (*n* = 247)^b^	participants without cancer (*n* = 224)	participants with any type of malignancy (*n* = 23)	
	median (P25, P75)	median (P25, P75)	median (P25, P75)	*P*-value^d^
TBARS (μM)	3.10 (1.75, 7.69)	3.10 (1.73, 7.61)	3.00 (1.95, 11.3)	0.585
SOD (U/mL)	9.03 (4.26, 16.1)	8.67 (4.31, 15.3)	13.7 (3.90, 24.5)	0.154
HO-1 (ng/mL)	17.5 (6.89, 24.3)	17.3 (6.75, 23.8)	20.3 (10.9, 27.7)	0.117
GPx (U/mL)	11.7 (8.41, 17.0)	11.7 (8.41, 16.8)	11.1 (8.58, 18.1)	0.602
GRd (U/mL)	0.10 (0.05, 0.17)	0.10 (0.05, 0.17)	*0.15 (0.09, 0.23)*	0.030
total glutathione (nmol/mL)	16.8 (3.90, 33.8)	17.3 (4.44, 35.1)	7.46 (0.01, 28.8)	0.228
GSSG (nmol/mL)	1.15 (0.00, 9.97)	1.45 (0.00, 9.97)	0.00 (0.00, 2.25)	0.282
GSH (nmol/mL)	8.50 (2.05, 21.8)	9.03 (2.34, 21.1)	6.17 (0.00, 24.3)	0.248
GSSG/GSH	0.41 (0.00, 1.00)	0.41 (0.00, 1.00)	0.41 (0.00, 1.00)	0.981
8OHdG^c^ (ng/mL)	0.54 (0.10, 1.72)	0.55 (0.15, 0.61)	0.45 (0.06, 1.72)	0.489

a Data are presented as median (percentile 25, percentile 75). Oxidative biomarkers: thiobarbituric acid reactive substances (TBARS); superoxide dismutase (SOD); heme oxygenase-1 (HO-1); glutathione peroxidase (GPx); glutathione reductase (GRd); oxidized glutathione (GSSG); reduced glutathione (GSH), 8-hydroxydeoxyguanosine.

b A total of 247 participants with oxidative stress biomarkers measured in AT after excluding benign tumors and basal cell carcinomas (BCCs). During the 16-year follow-up period, 224 participants remained free of any type of tumor and 23 developed any type of malignancy.

c Measurement only available in 209 study participants.

d *P*-value for the comparison between cancer and non-cancer cases using Mann–Whitney’s *U* test.

### 3.2 Longitudinal Associations between the AT Oxidative Microenvironment and Cancer Incidence

Table 4 presents the Cox regression analyses of the associations between ln-transformed AT oxidative biomarker levels and the risk of cancer. Among the oxidative biomarkers assessed, the effect estimates for SOD and GRd pointed toward a positive association with the risk of total cancer, although the confidence intervals included the null value [HRs (95% CIs): 1.38 (0.98, 1.93) and 1.43 (0.96, 2.12), respectively]. However, when only NHD tumors were considered, SOD and GRd levels were positively and significantly associated [HRs (95% CIs): 1.76 (1.17, 2.64) and 2.35 (1.41, 3.94), respectively] (Table 4). Although limited by a smaller number of cases, no associations were observed between any oxidative stress marker and HD cancer.

**Table 4:** Cox-Regression Analyses Showing Longitudinal Associations between AT Oxidative Stress Biomarkers and the 16-Year Cancer Incidence in the GraMo Cohort (*n* = 247)^a^

	total cancer incidence^b^			NHD cancers^c^			HD cancers^d^		
biomarker	HR (95% CI)	*p*-value	*n*/*N*	HR (95% CI)	*p*-value	*n*/*N*	HR (95% CI)	*p*-value	*n*/*N*
**SOD**	**1.38 (0.98, 1.93)**	**0.063**	**23/224**	**1.76 (1.17, 2.64)**	**0.007**	**17/224**	0.62 (0.27, 1.39)	0.243	6/224
HO-1	1.36 (0.79, 2.35)	0.267	23/224	1.24 (0.67, 2.29)	0.489	17/224	2.38 (0.67, 8.49)	0.181	6/224
GPx	1.05 (0.64, 1.72)	0.847	23/224	1.11 (0.56, 2.22)	0.761	17/224	0.94 (0.43, 2.09)	0.887	6/224
**GRd**	**1.43 (0.96, 2.12)**	**0.082**	**23/224**	**2.35 (1.41, 3.94)**	**0.001**	**17/224**	0.70 (0.39, 1.26)	0.230	6/224
total glutathione	0.93 (0.82, 1.04)	0.213	23/224	0.91 (0.79, 1.04)	0.161	17/224	0.99 (0.77, 1.27)	0.925	6/224
GSSG	0.95 (0.85, 1.06)	0.355	23/224	0.90 (0.78, 1.04)	0.171	17/224	1.03 (0.83, 1.26)	0.803	6/224
GSH	0.92 (0.83,1.03)	0.163	23/224	0.91 (0.80, 1.04)	0.160	17/224	0.96 (0.75, 1.21)	0.708	6/224
GSSG/GSH	1.00 (0.90, 1.13)	0.878	23/224	0.98 (0.86, 1.13)	0.818	17/224	1.07 (0.83, 1.38)	0.611	6/224
TBARS	1.16 (0.74, 1.82)	0.519	23/224	1.40 (0.80, 2.44)	0.240	17/224	0.77 (0.32, 1.85)	0.556	6/224
8OHdG^e^	0.94 (0.78, 1.14)	0.547	23/186	1.00 (0.79, 1.25)	0.977	17/186	0.83 (0.57, 1.19)	0.311	6/186

a Data are presented as hazard ratio and 95% confidence intervals [HR (95% CIs)]. Models were adjusted for age (years), sex (male/female), BMI (kg/m^2^), smoking (yes/no), alcohol consumption (yes/no), place of residence (urban vs semi-rural), and education (lower than primary education, primary education, or higher than primary). Oxidative biomarkers: thiobarbituric acid reactive substances (TBARS); superoxide dismutase (SOD); heme oxygenase (HO-1); glutathione peroxidase (GPx); glutathione reductase (GRd); oxidized glutathione (GSSG); reduced glutathione (GSH).

b All incident cases of cancer (*n* = 23), excluding benign tumors and basal cell carcinomas (BCCs). Rest of the study population (*n* = 224).

c Non-hormone-dependent (NHD) cancers (*n* = 17), excluding hormone-dependent cancers (*n* = 6) from the analysis. Rest of the study population (*n* = 224).

d Hormone-dependent cancers (*n* = 6), excluding non-hormone-dependent cancers (*n* = 17) from the analysis. Rest of the study population (*n* = 224).

e 8OHdG measures were only available for 209 participants.

### 3.3 Longitudinal Associations between POPs and Cancer Incidence

Table 5 displays the Cox regression analyses of the associations between AT ln-transformed POP concentrations and the risk of cancer. When all cancer cases were considered, β-HCH and PCB-138 were positively and significantly associated [HRs (95% CIs): 1.34 (1.00, 1.80) and 1.35 (1.00, 1.84), respectively]. Potential positive associations with total cancer were also observed for HCB (HR 1.26; 95% CI: 0.95, 1.66) and *p*,*p*′-DDE (HR 1.29; 95% CI: 0.96, 1.72). Exclusion of HD tumors strengthened all the previous associations (Table 5). Thus, PCB-138 was significantly and positively associated with NHD cancer (HR 1.78; 95% CI: 1.03, 3.09), while PCB-153 showed a positive borderline association (HR 1.80; 95% CI: 0.94, 3.47). Additionally, the organochlorine compounds HCB and β-HCH were also significantly and positively associated with the risk of NHD cancers [HRs (95% CI): 1.54 (1.02, 2.33) and 1.70 (1.09, 2.64), respectively] (Table 5). Participants with detectable α-HCH concentrations showed a marginally significant higher risk of NHD cancer (HR 2.79; 95% CI: 0.85, 9.11).

**Table 5:** Cox-Regression Analyses Showing Longitudinal Associations between AT Levels of POPs and the 16-Year Cancer Incidence in the GraMo Cohort (*n* = 348)^a^^,^^e^

	total cancer incidence^b^			NHD cancers^c^		
POPs	HR (95% CI)	*p*-value	*n*/*N*	HR (95% CI)	*p*-value	*n*/*N*
PCB-138	**1.35 (1.00, 1.84)**	**0.054**	**44/304**	**1.78 (1.03, 3.09)**	**0.038**	**27/304**
PCB-153	1.20 (0.84, 1.71)	0.313	44/304	1.80 (0.94, 3.47)	0.078	27/304
PCB-180	1.28 (0.90, 1.82)	0.175	44/304	1.56 (0.84, 2.87)	0.158	27/304
*p*,*p*′-DDE	1.29 (0.96, 1.72)	0.090	44/304	1.38 (0.94, 2.02)	0.098	27/304
HCB	1.26 (0.95, 1.66)	0.112	44/304	**1.54 (1.02, 2.33)**	**0.042**	**27/304**
β-HCH	**1.34 (1.00, 1.80)**	**0.053**	**44/304**	**1.70 (1.09, 2.64)**	**0.019**	**27/304**
α-HCH^d^	1.65 (0.69, 3.96)	0.265	44/304	2.79 (0.85, 9.11)	0.089	27/304
dicofol^d^	0.68 (0.29, 1.59)	0.378	44/304	1.17 (0.42, 3.28)	0.763	27/304

a Data are presented as hazard ratio and 95% confidence intervals [HR (95% CIs)]. Models were adjusted for age (years), sex (male/female), BMI (kg/m^2^), smoking (yes/no), alcohol consumption (yes/no), place of residence (urban vs semi-rural), and education (lower than primary education, primary education, or higher than primary).

b All incident cases of cancer (*n* = 44), excluding benign tumors and basal cell carcinomas (BCCs). Rest of the study population (*n* = 304).

c Non-hormone-dependent (NHD) cancers (*n* = 27), excluding hormone-dependent (HD) cancers (*n* = 17). Rest of the study population (*n* = 304).

d Participants with concentrations above the LOD were compared to those with non-detected concentrations.

e Note: no associations were observed between oxidative stress biomarkers and HD cancers in Table 4. Therefore, HD tumors were excluded from this analysis onward, given that we aimed to explore POP-cancer associations that could be mediated by *in situ* oxidative stress.

### 3.4 Mediation Analyses

Table 6 shows the results of the mediation analyses focused on the specific oxidative stress markers and POPs previously associated with the risk of NHD cancer (Tables 4 and 5, respectively). In line with Cox regression models, mediation models showed a significant total effect of PCB-138, β-HCH, and HCB on the risk of NHD cancer (Table 6). We observed positive indirect (i.e., mediated) effects for all the pollutants examined (PCB-138, HCB, and β-HCH), although confidence intervals included the null value. Although our limited sample size hampers the achievement of confidence intervals within the conventional statistical significance cutoff points, the indirect effect of β-HCH mediated by SOD and GRd was especially suggestive of a potential mediation [HR (95% CI): 1.10 (0.88, 1.39); percent mediated = 33%] and [HR (95% CI): 1.14 (0.90, 1.47); percent mediated = 47%], respectively (Table 6).

**Table 6:** Mediation Analysis^a^^,^^b^

oxidative stress marker	POPs	indirect effect HR (95% CI)^c^	direct effect HR (95% CI)^c^	total effect HR (95% CI)^c^	estimated percent mediated (%)^d^
SOD	HCB	1.07 (0.85, 1.33)	1.09 (1.01, 1.21)	1.17 (0.95, 1.44)	43
	β-HCH	**1.10 (0.88, 1.39)**	**1.22 (1.09, 1.40)**	**1.34 (1.09, 1.66)**	33
	PCB-138	1.04 (0.78, 1.39)	1.60 (1.29, 1.89)	1.66 (1.21, 1.85)	8
GRd	HCB	1.01 (0.79, 1.31)	1.19 (1.08, 1.36)	1.20 (0.98, 1.49)	6
	β-HCH	**1.14 (0.90, 1.47)**	**1.16 (1.05, 1.32)**	**1.32 (1.07, 1.65)**	47
	PCB-138	1.02 (0.81, 1.27)	1.05 (1.00, 1.12)	1.07 (0.86, 1.33)	29

a Effect estimates (95% CIs) of each natural log-unit increase in AT POP concentrations and the estimated percentage mediated by selected *in situ* oxidative stress biomarkers on the risk of non-hormone-dependent cancers (*n* = 247).

b Non-hormone-dependent (NHD) cancers (*n* = 17), excluding hormone-dependent cancers (*n* = 6) from the analysis. Rest of the study population (*n* = 224). Superoxide dismutase (SOD); glutathione reductase (GRd). Models were adjusted for age (years), sex (male/female), BMI (kg/m^2^), smoking (yes/no), alcohol consumption (yes/no), place of residence (urban vs semi-rural), and education (lower than primary education, primary education, or higher than primary).

c The direct effect, indirect effect, and total effect reflect the natural log hazard ratios (HR) and 95% confidence intervals (95% CI). The indirect effect represents the mediated effect.

d Percent mediated = indirect effect/(direct effect + indirect effect) × 100.

### 3.5 Sensitivity Analyses

No significant interactions were found for age, sex, BMI, education, place of residence, smoking habit, or alcohol consumption on the POP-cancer and oxidative stress-cancer associations. Sex-stratified models showed that the incidence of NHD cancer was higher in males compared to females, although associations were in the same direction and of a similar magnitude in both groups (Supporting Information, Table S5). When BMI was excluded from the models, effect estimates for POP-cancer associations were strengthened (Supporting Information Table S6). In addition to the chemicals significantly associated with NHD cancer in the main models (PCB-138, HCB, and β-HCH), PCB-153 and *p*,*p*′-DDE were also positively and significantly associated with NHD cancer [HRs (95% CIs): 2.03 (1.03, 4.00) and 1.49 (1.03, 2.14), respectively] (Supporting Information Table S6). Analogously, exclusion of BMI in mediation analyses slightly strengthened indirect effects (Supporting Information Table S7). Further adjustment of Cox-regression models (Tables 4 and 5) for the reason for surgery did not substantially change the main associations observed (Supporting Information Tables S8 and S9).

## 4 Discussion

Our results in adults from Southern Spain suggest that redox alterations in AT may be an early predictor of future cancer risk, especially AT levels of SOD and GRd in relation to the risk of total cancer and NHD tumors. Accumulated concentrations of PCB-138, β-HCH, and HCB in AT were also positively associated with the risk of developing NHD tumors. Moreover, mediation analyses suggested that the observed association between β-HCH and NHD cancer might be partially mediated by increased *in situ* SOD and GRd levels.

Although solid and mounting evidence highlights the important role of AT on cancer etiology and aggressiveness,^6^ to our best knowledge, no previous cohort study has investigated the relationship between AT’s oxidative microenvironment and cancer risk. Our results are consistent with the growing evidence proposing oxidative stress as one of the critical factors linking obesity with its associated chronic comorbidities.^7,73^

Our previous findings showed that POP mixtures in AT (especially β-HCH and PCBs) appeared to favor the Fenton reaction, increasing SOD activity and TBARS (lipid peroxidation) levels.^15^ Our present results suggest that increased oxidative stress levels in AT (increased SOD and GRd activities) could promote tumor development through the overgeneration of ROS/RNS radicals that cannot be compensated by antioxidants systems.^10^ Taken together, we hypothesize that increased POP concentrations in AT, especially β-HCH levels, could promote a low-grade chronic local oxidative state, increasing the chances of carcinogenic processes over time. This non-specific mode of action appears coherent with the heterogeneous group of NHD tumors investigated in this work.

The relationship between human POP exposure and specific cancer locations has been previously investigated, although no clear consensus has been reached. However, most studies have relied on case-control designs instead of prospective analyses.^44,48,74^ Exposure assessment is another important limitation: most studies have assessed POP concentrations in serum, which can be affected by point exposures and lifestyle modifications as well as by the so-called disease progression bias.^75,76^ On the contrary, AT is a more stable biological matrix that can better reflect the accumulated exposure to POPs over longer periods, which is of outermost importance when studying outcomes with a long latency period.^13,17,49^ Preliminary findings in the GraMo cohort found that accumulated PCB-153 concentrations were associated with total cancer risk, although the number of cancer cases was very limited at that time.^33^ In the present analysis, with a considerably higher follow-up time and a number of cancer cases more than doubled, not only PCBs (−138 and −153) but also the OCPs β-HCH and HCB were positively associated with the risk of NHD tumors.

Understanding causal mechanisms in observational studies is challenging and even more difficult given the complexity and long latency period of cancer. Although mediation analysis has been applied in social and epidemiological research for decades to understand causal pathways and biological mechanisms,^77^ surprisingly, its use in environmental and exposure epidemiology has been relatively infrequent.^13,78−81^ Although the results of our mediation analyses were not significant at a 95% CI cutoff point and should be interpreted with caution, they suggested that the potential long-term carcinogenic effect of β-HCH exposure might be partially mediated by alterations in local redox balance. This might also be the case for other highly correlated POPs, such as HCB and PCBs, although confidence intervals were larger and more imprecise. Of note, the mediation analyses performed were guided by previous results between oxidative stress and POPs with the outcome, thus reducing the possibility of chance findings. Given the novelty of our results, further confirmation is warranted in other populations as well as in a future follow-up of the GraMo cohort with a higher number of cancer cases. When enough incident HD cases occur in GraMo, associations of POP exposure with the risk of HD tumors will be analyzed, investigating hormonal biomarkers as potential mediators.

The carcinogenic effects of POPs are thought to occur *via* hormonal and non-hormonal mechanisms.^18^ Although epidemiological efforts have been focused on HD tumors,^44,48,74^ other authors have suggested potential non-endocrine action mechanisms, including increases in ROS generation and reactive nitrogen species through the induction of cytochrome P450,^82^ mitochondrial dysfunction,^30^ and/or inflammation.^83^ Based on the *in vitro* and *in vivo* evidence, non-dioxin-like PCBs induce the production of ROS, activate NF-κB transcription factors, and inhibit intercellular communication, all of which play a significant role in tumor promotion and progression.^3,84−86^ Lindane and its metabolites α-HCH and β-HCH have also been shown to induce immunosuppression and oxidative stress in both experimental animals, human cell lines, and occupational settings.^87^ Thus, markers of oxidative stress (e.g., SOD, catalase, TBARS, and GPx) were increased in human blood samples obtained from lindane poisoning cases in India, while glutathione levels were decreased.^88^ Even when POP mechanisms are complex and not fully elucidated, the mediation analysis conducted in this study is biologically plausible.^3,87^

Among the limitations of the present study is the modest sample size, especially in relation to cancer subgroups. As such, these results should be carefully interpreted. The hospital-based nature of our cohort may limit external validity, although this allowed the collection of AT samples from hundreds of participants. Indeed, GraMo constitutes the largest existing cohort of these characteristics and one of the few longitudinal cohorts studying this topic. The 16 years of follow-up adds plausibility to the study of associations with cancer given its particularly long-latency period. Our study focused not only on a hard endpoint such as cancer but also on subclinical mediators of disease, which reinforces the observed associations and the hypothesized causal pathways. A limitation of our mediation approach was that both the exposure and the mediator were measured in AT samples collected at baseline. However, while POP levels represent accumulated concentrations over long periods of time (even years),^49^ oxidative biomarkers probably reflect the redox state of AT around the time of sampling or at least a narrower time period than POPs.^58^ As a result, we do not expect a substantial alteration in the causal ordering of the exposure, mediator, and outcome.

The diversity of tumors represents a challenge. Thus, if the observed associations would only exist for certain cancer locations, the consideration of total cancer as the main outcome would bias our results to the null, rather than to false-positive associations. This is coherent with the fact that most borderline associations observed with total cancer were strengthened when NHD cancers were specifically considered. An alternative explanation for the associations found might be that obese people tend to accumulate more POPs due to their lipophilic properties. Under this view, our POP-cancer associations could also be explained in terms of non-causal relationships. Although this possibility exists, the main models were adjusted for BMI, which can partially account for differences in adiposity. Moreover, the potential mediation of oxidative stress between the β-HCH-cancer association and the longitudinal design adds weight against this alternative explanation. Finally, given that POPs constitute a very large family of chemicals with similar physicochemical characteristics, we cannot rule out that the observed associations might also be surrogates of other highly correlated and unmeasured co-exposures, such as other PCBs, polybrominated diphenyl ethers, or dioxin-like compounds,^89,90^ or even a surrogate of correlated chemical mixtures acting in a combined manner.^91,92^

In conclusion, our findings highlight the importance of the human AT redox microenvironment as an early predictor of future cancer development and suggest that POP accumulation in AT might alter this *in situ* redox balance, leading to an increased risk of NHD cancer. Given the economic and societal costs of cancer, environmental and public health interventions are needed to progressively reduce the accumulated POP body burden to protect current and future generations.

## Supporting Information Available

Additional descriptive analysis of study participants; localization and classification of benign tumors; sex-stratified descriptive analysis; sex-stratified POP-cancer associations; sensitivity analysis of main models without BMI adjustment; and sensitivity analysis of main models further adjusted for reason for surgery (PDF)

## Notes

This paper will form part of the doctoral thesis developed by Francisco M. Pérez-Carrascosa in the context of the “Clinical Medicine and Public Health Program” of the University of Granada (Spain).
