# Supplementary material for: Adipose Tissue Redox Microenvironment as a Potential Link between Persistent Organic Pollutants and the 16-Year Incidence of Non-hormone-Dependent Cancer
Source: Environ Sci Technol. 2021 Jun 28;55(14):9926–37. doi: 10.1021/acs.est.0c08180 (PMC8474112; doi:10.1021/acs.est.0c08180)
Supplement: Supplementary file 1 — es0c08180_si_001.pdf [file es0c08180_si_001.pdf]

**Supporting Information**

## Adipose tissue redox microenvironment as a potential link between persistent organic pollutants and the 16-year incidence of non-hormone dependent cancer

Vicente Mustieles, Francisco M. Pérez-Carrascosa, Josefa León, Theis Lange, Jens-Peter Bonde, Celia Gómez-Peña, Francisco Artacho-Cordón, Rocío Barrios-Rodríguez, Rocío Olmedo-Requena, José Expósito, José J. Jiménez-Moleón, Juan P. Arrebola

**Supplemental Table 1. Comparison of sociodemographic and exposure characteristics between participants with both adipose tissue POPs and oxidative stress (OS) biomarkers (n=247) and those with POPs but without OS (n=101).**

| | POPs + OS (n=247) | | Only POPs (n=101) | | P-value [a] |
|---|---|---|---|---|---|
| | n | % | n | % | |
| **Sex= male** | **134** | **54.3** | **41** | **40.6** | **0.018** |
| **Education** | | | | | 0.327 |
| Primary uncompleted | 67 | 27.1 | 28 | 27.7 | |
| Primary | 116 | 47.0 | 39 | 38.6 | |
| Secondary or higher | 64 | 25.9 | 34 | 33.7 | |
| **Residence** | | | | | 0.638 |
| Urban | 129 | 52.2 | 50 | 49.5 | |
| Semi-rural | 118 | 47.8 | 51 | 50.5 | |
| **Alcohol consumer (=yes)** | 126 | 51.0 | 55 | 54.5 | 0.724 |
| **Smoker (=yes)** | 79 | 40.0 | 34 | 33.7 | 0.900 |
| | **Median (P25, P75)** | | **Median (P25, P75)** | | |
| **Age (years)** | 51.0 (35.0, 64.0) | | 50.0 (37.5, 59.0) | | 0.338 |
| **BMI (kg/m$^2$)** | **27.1 (23.9, 29.8)** | | **25.7 (23.7, 28.0)** | | **0.034** |
| **PCB-138 (ng/g)** | 82.4 (35.2, 138.7) | | 80.7 (3.82, 132.4) | | 0.323 |
| **PCB-153 (ng/g)** | 216.9 (142.7, 356.2) | | 225.7 (105.4, 389.4) | | 0.764 |
| **PCB-180 (ng/g)** | 179.8 (107.8, 284.3) | | 173.0 (82.5, 301.9) | | 0.513 |
| ***p,p'*-DDE (ng/g)** | 96.4 (36.0, 211.9) | | 87.9 (27.9, 211.1) | | 0.523 |
| **HCB (ng/g)** | 14.0 (5.41, 40.1) | | 15.7 (4.53, 36.4) | | 0.633 |
| ***β*-HCH (ng/g)** | 10.0 (4.14, 21.3) | | 11.6 (1.35, 20.7) | | 0.978 |
| | n | % | n | % | |
| **Dicofol (>LOD)** | 52 | 21.1 | 19 | 18.8 | 0.842 |
| ***α*-HCH (>LOD)** | 51 | 20.7 | 19 | 18.8 | 0.769 |

[a] P-value for the comparison between cancer and non-cancer cases. Fisher's exact test and Mann-Whitney's U for categorical and continuous variables, respectively.

**Supplemental Table 2. Localization and classification of benign tumors.**

| | | ICD-10 | n | n % |
|---|---|---|---|---|
| Non Hormone-dependent | **Skin** | D23 | 8 | 30.77% |
| | **Peripheral nerves** | D48 | 2 | 7.69% |
| | **Nasopharynx** | D10 | 2 | 7.69% |
| | **Pancreas** | D13 | 1 | 3.85% |
| | **Soft tissues** | D21 | 1 | 3.85% |
| | **Eye** | D31 | 1 | 3.85% |
| | **Hematological** | D47 | 1 | 3.85% |
| Hormone - dependent | **Breast** | N60 | 3 | 11.54% |
| | **Prostate** | N40 | 3 | 11.54% |
| | **Uterus body** | D25 | 2 | 7.69% |
| | **Ovary** | N80 | 2 | 7.69% |
| **Total number of benign tumors** | | | 26 | 100% |

**Supplemental Table 3. Sex-stratified descriptive analysis of sociodemographic characteristics and adipose tissue POP concentrations (n=348).**

| | Men (n=175) | | Women (n=173) | | P-value [a] |
|---|---|---|---|---|---|
| | n | % | n | % | |
| **Education** | | | | | 0.533 |
| Primary uncompleted | 49 | 28.0 | 46 | 26.6 | |
| Primary | 73 | 41.7 | 82 | 47.4 | |
| Secondary or higher | 53 | 30.3 | 45 | 26.0 | |
| **Residence** | | | | | |
| Urban | **100** | **57.1** | **79** | **45.7** | **0.041** |
| Semi-rural | 75 | 42.9 | 94 | 54.3 | |
| **Alcohol consumer (=yes)** | **132** | **75.4** | **49** | **28.3** | **<0.001** |
| **Smoker (=yes)** | **75** | **42.9** | **38** | **22.0** | **<0.001** |
| | **Median (P25, P75)** | | **Median (P25, P75)** | | |
| **Age (years)** | 51.0 (35.0, 63.0) | | 50.0 (36.5, 63.0) | | 0.874 |
| **BMI (kg/m$^2$)** | 26.8 (24.4, 29.1) | | 26.2 (23.4, 29.8) | | 0.401 |
| **PCB-138 (ng/g)** | 72.9 (28.6, 123.3) | | 86.4 (36.3, 147.5) | | 0.206 |
| **PCB-153 (ng/g)** | 210.7 (126.5, 337.9) | | 234.5 (139.1, 383.0) | | 0.412 |
| **PCB-180 (ng/g)** | 179.0 (101.2, 309.4) | | 177.2 (104.4, 291.7) | | 0.750 |
| ***p,p'*-DDE (ng/g)** | **70.2 (28.8, 175.7)** | | **114.0 (38.6, 283.1)** | | **<0.001** |
| **HCB (ng/g)** | **9.4 (3.8, 24.2)** | | **28.4 (8.0, 53.3)** | | **<0.001** |
| ***β*-HCH (ng/g)** | **7.3 (2.3, 14.5)** | | **15.3 (6.6, 30.0)** | | **<0.001** |
| | **n** | **%** | **n** | **%** | |
| **Dicofol (>LOD)** | 36 | 20.6 | 35 | 20.2 | 1.000 |
| ***α*-HCH (>LOD)** | **20** | **11.4** | **50** | **28.9** | **<0.001** |

[a] P-value for the comparison between cancer and non-cancer cases. Fisher's exact test and Mann-Whitney's U for categorical and continuous variables, respectively.

**Supplemental Table 4. Sex-stratified descriptive analysis of oxidative stress biomarkers (n=247).**

| | Men (n=134) | Women (n=113) | P-value [a] |
|---|---|---|---|
| | **Median (P25, P75)** | **Median (P25, P75)** | |
| **TBARS (µM)** | 2.70 (1.58, 7.37) | 3.78 (1.88, 7.98) | 0.178 |
| **SOD (U/mL)** | 8.53 (3.95, 17.2) | 9.18 (4.93, 15.6) | 0.720 |
| **HO-1 (ng/ml)** | 18.9 (8.24, 24.5) | 13.7 (5.90, 23.9) | 0.112 |
| **GPx (U/mL)** | 11.8 (9.13, 17.1) | 11.2 (8.23, 16.8) | 0.453 |
| **GRd (U/mL)** | 0.09 (0.04, 0.16) | 0.11 (0.06, 0.18) | 0.120 |
| **Total glutathione (nmol/ml)** | 16.3 (3.20, 31.2) | 18.9 (4.90, 35.9) | 0.461 |
| **GSSG (nmol/ml)** | **0.34 (0.01, 8.15)** | **3.08 (0.01, 15.0)** | **0.037** |
| **GSH (nmol/ml)** | 9.47 (2.95, 24.5) | 7.54 (1.86, 20.5) | 0.394 |
| **GSSG/GSH** | **0.28 (0.01, 1.00)** | **0.64 (0.03, 1.23)** | **0.006** |
| **8OHdG [b] (ng/ml)** | 0.45 (0.09, 1.40) | 0.75 (0.25, 2.34) | 0.079 |

Data are presented as median (percentile 25, percentile 75). Oxidative biomarkers: thiobarbituric acid reactive substances (TBARS); superoxide dismutase (SOD); heme oxygenase-1 (HO-1); glutathione peroxidase (GPx); glutathione reductase (GRd); oxidized glutathione (GSSG); reduced glutathione (GSH), 8-hydroxydeoxyguanosine (8OHdG).

[a] P-value for the comparison between cancer and non-cancer cases using Mann-Whitney's U test.

[b] Measurement only available in 209 study participants (113 men and 96 women).

**Supplemental Table 5. Sex-stratified Cox-regression analyses showing longitudinal associations between adipose tissue levels of persistent organic pollutants and the 16-year incidence of total and non-hormone dependent cancers in GraMo cohort (n=348).**

| Women (n=173) | | | | | | |
|---|---|---|---|---|---|---|
| | **Total cancer incidence [a/c]** | | | **NHD cancers [b/d]** | | |
| **POPs** | **HR (95% CI)** | **p-value** | **n/N** | **HR (95% CI)** | **p-value** | **n/N** |
| **PCB-138** | **2.05 (1.05, 4.00)** | **0.035** | **21/152** | 2.05 (0.76, 5.55) | 0.159 | 10/152 |
| **PCB-153** | **2.02 (0.93, 4.30)** | **0.075** | **21/152** | 2.14 (0.69, 6.69) | 0.189 | 10/152 |
| **PCB-180** | **2.21 (1.03, 4.71)** | **0.041** | **21/152** | 1.76 (0.62, 4.98) | 0.284 | 10/152 |
| ***p,p'*-DDE** | 1.40 (0.91, 2.16) | 0.126 | 21/152 | 1.71 (0.85, 3.45) | 0.130 | 10/152 |
| **HCB** | **1.59 (0.95, 2.66)** | **0.080** | 21/152 | 1.79 (0.81, 3.93) | 0.148 | 10/152 |
| **β-HCH** | 1.34 (0.82, 2.20) | 0.247 | 21/152 | 1.73 (0.68, 4.39) | 0.249 | 10/152 |
| **α-HCH** | 1.39 (0.43, 4.53) | 0.588 | 21/152 | 4.39 (0.60, 32.2) | 0.146 | 10/152 |
| **Dicofol** | 0.46 (0.10, 2.13) | 0.322 | 21/152 | 0.51 (0.05, 4.80) | 0.556 | 10/152 |

| Men (n=175) | | | | | | |
|---|---|---|---|---|---|---|
| **POPs** | **HR (95% CI)** | **p-value** | **n/N** | **HR (95% CI)** | **p-value** | **n/N** |
| **PCB-138** | 1.15 (0.84, 1.58) | 0.392 | 23/152 | 1.62 (0.85, 3.07) | 0.142 | 17/152 |
| **PCB-153** | 0.92 (0.64, 1.34) | 0.670 | 23/152 | 1.42 (0.60, 3.36) | 0.427 | 17/152 |
| **PCB-180** | 0.94 (0.64, 1.38) | 0.759 | 23/152 | 1.15 (0.53, 2.49) | 0.723 | 17/152 |
| ***p,p'*-DDE** | 1.18 (0.80, 1.75) | 0.404 | 23/152 | 1.21 (0.77, 1.92) | 0.408 | 17/152 |
| **HCB** | 1.14 (0.81, 1.62) | 0.458 | 23/152 | **1.64 (1.00, 2.69)** | **0.052** | **17/152** |
| **β-HCH** | **1.40 (0.97, 2.03)** | **0.074** | 23/152 | **1.75 (1.08, 2.84)** | **0.023** | **17/152** |
| **α-HCH** | 2.35 (0.61, 9.14) | 0.217 | 23/152 | 1.55 (0.27, 9.02) | 0.628 | 17/152 |
| **Dicofol** | 0.75 (0.24, 2.36) | 0.622 | 23/152 | 1.56 (0.45, 5.43) | 0.486 | 17/152 |

Data are presented as Hazard Ratio and 95% Confidence Intervals [HR (95% CIs)]. Models were adjusted for age (years), sex (male/female), BMI (kg/m$^2$), smoking (yes/no), alcohol consumption (yes/no), place of residence (urban *vs.* semi-rural) and education (lower than primary education, primary education or higher than primary).

[a] Female incident cases of total cancer (n=21) excluding benign tumors and basal cell carcinomas (BCCs). Rest of the study population (n=152).
[b] Non-hormone dependent (NHD) cancers (n=10), excluding hormone-dependent (HD) cancers (n=11). Rest of the study population (n=152).
[c] Male incident cases of total cancer (n=23) excluding benign tumors and basal cell carcinomas (BCCs). Rest of the study population (n=152).
[d] Non-hormone dependent (NHD) cancers (n=17) excluding hormone-dependent (HD) cancers (n=6). Rest of the study population (n=152).

**Supplemental Table 6. Cox-regression analyses showing longitudinal associations between adipose tissue levels of persistent organic pollutants and the 16-year cancer incidence in GraMo cohort, without adjustment for BMI (n=348).**

| POPs | Total cancer incidence [a] | | | NHD cancers [b] | | |
|---|---|---|---|---|---|---|
| | HR (95% CI) | p-value | n/N | HR (95% CI) | p-value | n/N |
| **PCB-138** | **1.40 (1.03, 1.92)** | **0.034** | **44/304** | **1.97 (1.12, 3.46)** | **0.019** | **27/304** |
| **PCB-153** | 1.25 (0.86, 1.80) | 0.244 | 44/304 | **2.03 (1.03, 4.00)** | **0.040** | **27/304** |
| **PCB-180** | 1.30 (0.91, 1.87) | 0.148 | 44/304 | 1.65 (0.87, 3.12) | 0.124 | 27/304 |
| ***p,p'*-DDE** | **1.35 (1.01, 1.78)** | **0.040** | **44/304** | **1.49 (1.03, 2.14)** | **0.034** | **27/304** |
| **HCB** | **1.31 (1.00, 1.72)** | **0.052** | **44/304** | **1.65 (1.11, 2.45)** | **0.014** | **27/304** |
| **β-HCH** | **1.39 (1.04, 1.86)** | **0.027** | **44/304** | **1.79 (1.17, 2.74)** | **0.008** | **27/304** |
| **α-HCH [c]** | 1.77 (0.75, 4.20) | 0.196 | 44/304 | 3.01 (0.96, 9.39) | 0.058 | 27/304 |
| **Dicofol [c]** | 0.70 (0.30, 1.61) | 0.397 | 44/304 | 1.26 (0.46, 3.46) | 0.763 | 27/304 |

Data are presented as Hazard Ratio and 95% Confidence Intervals [HR (95% CIs)]. Models were adjusted for age (years), sex (male/female), smoking (yes/no), alcohol consumption (yes/no), place of residence (urban *vs.* semi-rural) and education (lower than primary education, primary education or higher than primary).

[a] All incident cases of cancer (n=44), excluding benign tumors and basal cell carcinomas (BCCs). Rest of the study population (n=304).
[b] Non-hormone dependent (NHD) cancers (n=27), excluding hormone-dependent (HD) cancers (n=17). Rest of the study population (n=304).
[c] Participants with concentrations above the limit of detection were compared to those with non-detected concentrations.

**Supplemental Table 7. Mediation analysis. Effect estimates (95% CIs) of each natural log-unit increase in adipose tissue POP concentrations and the estimated percentage mediated by selected in situ oxidative stress biomarkers on the risk of non-hormone dependent cancers, without adjustment for BMI (n=247).**

| Oxidative Stress marker | POPs | Indirect effect HR (95% CI) [a] | Direct effect HR (95% CI) [a] | Total effect HR (95% CI) [a] | Estimated percent mediated (%) [b] |
|---|---|---|---|---|---|
| **SOD** | **HCB** | 1.11 (0.87, 1.40) | 1.19 (1.07, 1.35) | 1.31 (1.07, 1.60) | 39 |
| | **β-HCH** | **1.14 (0.91, 1.44)** | **1.28 (1.14, 1.51)** | **1.47 (1.17, 1.81)** | 34 |
| | **PCB-138** | 1.09 (0.79, 1.46) | 1.61 (1.28, 1.96) | 1.76 (1.26, 1.96) | 15 |
| **GRd** | **HCB** | 1.05 (0.83, 1.33) | 1.28 (1.14, 1.52) | 1.35 (1.11, 1.65) | 16 |
| | **β-HCH** | **1.16 (0.92, 1.48)** | **1.24 (1.10, 1.45)** | **1.44 (1.15, 1.77)** | 41 |
| | **PCB-138** | 1.14 (0.83, 1.56) | 1.49 (1.24, 1.89) | 1.70(1.22, 2.05) | 25 |

Non-hormone dependent (NHD) cancers (n=17), excluding hormone-dependent cancers (n=6) from the analysis. Rest of the study population (n=224). Superoxide dismutase (SOD); glutathione reductase (GRd). Models were adjusted for age (years), sex (male/female), smoking (yes/no), alcohol consumption (yes/no), place of residence (urban *vs.* semi-rural) and education (lower than primary education, primary education or higher than primary).

[a] The direct effect, indirect effect, and total effect reflect the natural log Hazard Ratios (HR) and 95% Confidence Intervals (95% CI). The indirect effect represents the mediated effect.

[b] Percent mediated = indirect effect/ (direct effect + indirect effect) × 100.

**Supplemental Table 8. Cox-regression analyses showing longitudinal associations between adipose tissue oxidative stress biomarkers and the 16-year cancer incidence in GraMo cohort (n=247), with further adjustment for reason for surgery.**

| Biomarker | Total cancer incidence [a] | | | NHD cancers [b] | | |
|---|---|---|---|---|---|---|
| | HR (95% CI) | p-value | n/N | HR (95% CI) | p-value | n/N |
| **SOD** | **1.36 (0.97, 1.91)** | **0.074** | **23/224** | **1.76 (1.16, 2.67)** | **0.007** | **17/224** |
| **HO-1** | 1.40 (0.82, 2.37) | 0.213 | 23/224 | 1.34 (0.73, 2.45) | 0.340 | 17/224 |
| **GPx** | 1.08 (0.67, 1.77) | 0.747 | 23/224 | 1.13 (0.58, 2.19) | 0.720 | 17/224 |
| **GRd** | **1.37 (0.92, 2.06)** | **0.123** | **23/224** | **2.32 (1.34, 4.02)** | **0.003** | **17/224** |
| **Total Glutathione** | 0.94 (0.83, 1.05) | 0.270 | 23/224 | 0.91 (0.79, 1.05) | 0.188 | 17/224 |
| **GSSG** | 0.93 (0.83, 1.04) | 0.228 | 23/224 | 0.87 (0.75, 1.01) | 0.072 | 17/224 |
| **GSH** | 0.94 (0.84,1.05) | 0.258 | 23/224 | 0.92 (0.81, 1.05) | 0.228 | 17/224 |
| **GSSG/GSH** | 0.98 (0.88, 1.10) | 0.753 | 23/224 | 0.95 (0.83, 1.09) | 0.439 | 17/224 |
| **TBARS** | 1.15 (0.73, 1.79) | 0.549 | 23/224 | 1.37 (0.78, 2.38) | 0.271 | 17/224 |
| **8OHdG [c]** | 0.94 (0.78, 1.14) | 0.580 | 23/186 | 1.03 (0.80, 1.31) | 0.840 | 17/186 |

Data are presented as Hazard Ratio and 95% Confidence Intervals [HR (95% CIs)]. Models were adjusted for: age (years), sex (male/female), BMI (kg/m$^2$), smoking (yes/no), alcohol consumption (yes/no), place of residence (urban *vs.* semi-rural), education (lower than primary education, primary education or higher than primary) and reason for surgery (hernias, gallbladder disease, varicose veins and other conditions). Oxidative biomarkers: thiobarbituric acid reactive substances (TBARS); superoxide dismutase (SOD); heme oxygenase (HO-1); glutathione peroxidase (GPx); glutathione reductase (GRd); glutathione S-transferase (GST); oxidized glutathione (GSSG); reduced glutathione (GSH).

[a] All incident cases of cancer (n=23), excluding benign tumors and basal cell carcinomas (BCCs). Rest of the study population (n=224).
[b] Non-hormone dependent (NHD) cancers (n=17), excluding hormone-dependent cancers (n=6) from the analysis. Rest of the study population (n=224).
[c] 8OHdG measures were only available for 209 participants

**Supplemental Table 9. Cox-regression analyses showing longitudinal associations between adipose tissue levels of persistent organic pollutants and the 16-year cancer incidence in GraMo cohort (n=348), with further adjustment for reason for surgery.**

| POPs | Total cancer incidence [a] | | | NHD cancers [b] | | |
|---|---|---|---|---|---|---|
| | HR (95% CI) | p-value | n/N | HR (95% CI) | p-value | n/N |
| **PCB-138** | **1.31 (0.96, 1.79)** | **0.093** | **44/304** | **1.73 (0.99, 3.01)** | **0.053** | **27/304** |
| **PCB-153** | 1.13 (0.79, 1.62) | 0.493 | 44/304 | 1.68 (0.86, 3.29) | 0.132 | 27/304 |
| **PCB-180** | 1.23 (0.85, 1.76) | 0.274 | 44/304 | 1.45 (0.78, 2.70) | 0.237 | 27/304 |
| ***p,p'*-DDE** | 1.16 (0.87, 1.56) | 0.312 | 44/304 | 1.31 (0.89, 1.94) | 0.172 | 27/304 |
| **HCB** | 1.27 (0.94, 1.71) | 0.125 | 44/304 | **1.54 (1.01, 2.35)** | **0.045** | **27/304** |
| **β-HCH** | **1.31 (0.97, 1.77)** | **0.074** | **44/304** | **1.64 (1.06, 2.54)** | **0.025** | **27/304** |
| **α-HCH [c]** | 1.49 (0.62, 3.63) | 0.376 | 44/304 | 2.78 (0.84, 9.25) | 0.096 | 27/304 |
| **Dicofol [c]** | 0.77 (0.32, 1.81) | 0.542 | 44/304 | 1.22 (0.44, 3.39) | 0.707 | 27/304 |

Data are presented as Hazard Ratio and 95% Confidence Intervals [HR (95% CIs)]. Models were adjusted for age (years), sex (male/female), BMI (kg/m$^2$), smoking (yes/no), alcohol consumption (yes/no), place of residence (urban *vs.* semi-rural), education (lower than primary education, primary education or higher than primary) and reason for surgery (hernias, gallbladder disease, varicose veins and other conditions).

[a] All incident cases of cancer (n=44), excluding benign tumors and basal cell carcinomas (BCCs). Rest of the study population (n=304).
[b] Non-hormone dependent (NHD) cancers (n=27), excluding hormone-dependent (HD) cancers (n=17). Rest of the study population (n=304).
[c] Participants with concentrations above the limit of detection were compared to those with non-detected concentrations.
